# Bonding Performance of Universal Adhesives Applied to Nano-Hydroxyapatite Desensitized Dentin Using Etch-and-Rinse or Self-Etch Mode

**DOI:** 10.3390/ma14164746

**Published:** 2021-08-22

**Authors:** Yuchen Meng, Fan Huang, Silin Wang, Meiwen Li, Yi Lu, Dandan Pei, Ang Li

**Affiliations:** 1 Key Laboratory of Shaanxi Province for Craniofacial Precision Medicine Research, College of Stomatology, Xi’an Jiaotong University, Xi’an 710004, China; mengyuchen@stu.xjtu.edu.cn (Y.M.); nimbus2015@stu.xjtu.edu.cn (S.W.); lmw1916273766@stu.xjtu.edu.cn (M.L.); drliang@mail.xjtu.edu.cn (A.L.); 2 Department of Prosthodontics, College of Stomatology, Xi’an Jiaotong University, Xi’an 710004, China; frank616@stu.xjtu.edu.cn (F.H.); luyi1962@xjtu.edu.cn (Y.L.)

**Keywords:** universal adhesive, etch-and-rinse mode, self-etch mode, nano-hydroxyapatite, dentin hypersensitivity

## Abstract

The study assessed the bonding performance of three universal adhesives on desensitized dentin with etch-and-rinse mode or self-etch mode after nano-hydroxyapatite (nHAp)-based desensitizers application. Simulated sensitive dentin specimens were prepared and separated into four groups: no treatment as the negative control, groups desensitized by Biorepair toothpaste, Dontodent toothpaste, or nHAp paste. Three universal adhesives of All-Bond Universal, Single Bond Universal, and Clearfil Universal Bond with etch-and-rinse or self-etch mode were bonded to the desensitized dentin specimens separately, followed by resin composite build-ups. Micro-tensile bond strength was measured using a micro-tensile tester. The wettability of desensitized dentin was evaluated by the contact angle of the adhesives. Resin infiltration was observed by confocal laser scanning microscopy. Dentin tubular occlusion and nanoleakage were observed by scanning electron microscope. The results showed that the etch-and-rinse or self-etch mode of each adhesive showed similar bond strength when bonding to nHAp-based desensitized dentin. The dentin surface was partially covered by desensitizers after desensitization. Compared with the self-etch mode, stronger demineralization and more reopened dentin tubules were observed in the etch-and-rinse mode after acid etching; longer resin tags and more nanoleakage in the resin–dentin interface were observed when using the etch-and-rinse mode. When bonding to nHAp-based desensitized dentin with universal adhesives, no significant difference in bond strength was found between self-etch mode or etch-and-rinse mode; while the latter produced more nanoleakage in the resin–dentin interfaces.

## 1. Introduction

Dentin adhesive systems are categorized into two bonding modes, etch-and-rinse and self-etch [1]. Mechanical interlocking between resin and dentin contributes to the primary bond strength. Both bonding modes have their own particularities in the bonding mechanism. In etch-and-rinse mode, phosphoric acid is used to remove the smear layer completely, resulting in micron-level demineralization of dentin to allow the subsequent resin monomer infiltration [2]. In self-etch mode, acidic monomers pre-incorporated into adhesives replace the separate phosphoric acid etching procedure, creating a hybrid layer mixed with residual smear layer on a moderate demineralized surface [3]. In recent years, the newest universal adhesives have been much welcomed by dentists due to their simplicity, multimodality, and user-friendliness. They can satisfy both bonding modes and various substrates with favorable bond strength, owing to the specific functional monomers in adhesive systems such as silane and 10-methacryloyloxydecyl dihydrogen phosphate (10-MDP). Meanwhile, the functional monomers can provide additional chemical interaction with dentin substrate [3,4,5,6].

Dentin hypersensitivity (DH) is a frequent oral disease [7,8]. Through exposed dentin of the sensitive tooth, external stimulus initiates rapid fluid flow within dentinal tubules which are patent from pulp, and triggers nerve responses of pain sensation [9,10,11], causing transient but terrible suffering to the patients. The main therapeutic strategies include: (1) stabilizing the nerve and block nerve response in the pulp; (2) occluding the exposed dentinal tubules and preventing the fluid flow in the tubules [9,12]; the latter have achieved broad success in DH relief [7,13,14,15]. According to the handbook of treating dentin hypersensitivity, home-use desensitizing toothpaste is considered as the initial choice for mild discomfort or pain [9]. As one of the most biocompatible and bioactive materials, nano-hydroxyapatite (nHAp) can form superior tubular occlusion to establish a mechanical barrier attributed to its nano-sized particles analogous with tooth apatite crystals. Therefore, nHAp-based desensitizing toothpastes have been praised among these home-use therapies [16,17,18,19].

In clinical practice, DH always occurs as a result of tooth defects. Restorative intervention is recognized as a follow-up complementary therapy to repair the tooth shape and provide effective, long-lasting alleviation after applying desensitizing agents [20,21]. Direct composite resin and dentin bonding systems are indicated for the situation. According to the properties of the bonding substrate, it is important to choose the appropriate adhesive and bonding mode to make sure the quality of the resin–dentin bonding in clinical practice. Different desensitizers showed various effects on resin–dentin bond strengths based on a meta-analysis. Except for casein phosphopeptide-amorphous calcium phosphate (CPP-ACP) and arginine-CaCO_3_, most other desensitizers negatively affected bond strength. Meanwhile, various adhesives presented significant differences in dentin bond strength [22]. When using self-etch adhesive systems after applying nHAp-based desensitizing toothpastes, the bond strength decreased due to resin infiltration interfered by tight dentin tubular sealing as demonstrated by our previous studies [23]. Consistent conclusions were also verified by Arisu et al. and Makkar et al. on self-etch adhesives [24,25]. However, other studies observed no decline in bond strength when using etch-and-rinse adhesive systems on dentin surface pretreated with desensitizers [26,27,28]. At present, researches of universal adhesives on desensitized dentin surface are limited to the self-etch mode. Zhang et al. and Chiang et al. selected Single Bond Universal as a representative adhesive for experiments and obtained similar results that the desensitizing agents had no adverse effect on the bond strength [13,29]. However, for universal adhesives, it is unclear whether self-etch mode or etch-and-rinse mode can obtain preferable bonding performance on the desensitized dentin after nHAp-based desensitizers application.

The present study aimed to evaluate three universal adhesives’ bonding performance with etch-and-rinse mode or self-etch mode after applying nHAp-based desensitizing agents. The null hypotheses tested were: bonding modes would not affect adhesive parameters of universal adhesives on the desensitized dentin, including bond strength, dentin tubular occlusion, contact angle, and adhesive interface observation.

## 2. Materials and Methods

### 2.1. Sensitive Specimen Preparation

**Figure 1:**
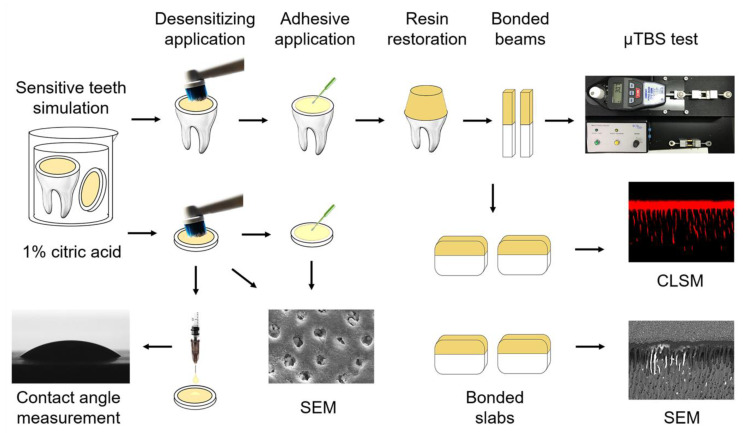
Schematic illustration of the study design. Mid-coronal and disc specimens were prepared for sensitive models and applied by desensitizing treatments. Mid-coronal specimens were bonded with universal adhesives, restored by resin composite, then sectioned into beams and slabs. μTBS were tested on bonded beams. Adhesive interfaces were observed on bonded slabs by CLSM and SEM. Dentin tubular occlusion before and after adhesive application were analyzed by SEM. Wettability of universal adhesives was measured by the contact angle.

After approval by the Ethics Committee of School & Hospital of Stomatology, Xi’an Jiaotong University (approved number xjkqll [2018]030), 300 human third molars without caries were obtained and stored in 0.02 wt % NaN_3_ solution at 4 °C. Enamel and shallow dentin were cut out perpendicularly to the longitudinal axis by a low-speed diamond saw (Isomet, Buehler, Lake Bluff, IL, USA) under water coolant to prepare mid-coronal and disc specimens. All exposed dentin surfaces were polished with wet 600-grit silicon carbide paper for 1 min and then soaked in a 1 wt % citric acid solution for 20 s to imitate sensitive models [30]. The schematic of the experimental design is shown in Figure 1.

### 2.2. Surface Desensitization

Before adhesive application, all samples were randomly assigned into 4 groups and received desensitizing treatment as follows: no desensitization (control); Biorepair toothpaste (Dr. K.Wolff GmbH, Bielefeld, Germany) was brushed over dentin surface with an electric toothbrush (model 3756, Oral B, Cincinnati, OH, USA) for 2 min, and then reposed for 3 min before water-spray rinse; Dontodent Sensitive toothpaste (DM-Drogerie Markt, Karlsruhe, Germany), and experimental nHAp paste (Sigma-Aldrich, St. Louis, MO, USA) mixed by nanopowder and deionized water in a 1:1 mass ratio were utilized the same way as Biorepair. During the interval of desensitization, the specimens were stored at 37 °C in artificial saliva [31].

### 2.3. Bonding Procedure

Three universal adhesives, All-Bond Universal, Single Bond Universal and Clearfil Universal Bond, were tested in the study. The manufacturers, compositions, and application procedures for etch-and-rinse and self-etch modes were in Table 1. After desensitization, 192 mid-coronal dentin specimens were randomly grouped and bonded using three universal adhesives with either etch-and-rinse mode or self-etch mode according to the manufacturer’s instructions, separately (*n* = 8 per group). For etch-and-rinse mode, dentin surfaces were etched with 35% H_3_PO_4_ (Gluma Etchant, Heraeus Kulzer, Hanau, Germany) for 15 s, followed by thorough rinsing and gentle drying to generate a visible moist surface with slight air blowing. Then, adhesives were applied on the dentin surface using a micro brush and light-activated by a light-curing unit (Sirona Dental, Bensheim, Germany) for 10 s at 1200 mW/cm^2^ output density. Next, four layers of 1-mm-thick resin composite (Filtek Z-250, 3M ESPE, St. Paul, MN, USA) were placed and light-cured for 20 s.

### 2.4. Micro-Tensile Bond Testing (μTBS)

Each bonded sample was sliced into 0.9-mm-thick slabs vertically to the bonding interface after 24 h storage. The central slabs were selected for nanoleakage observation, and the others were further cut into 0.9 mm × 0.9 mm sticks. Five longest sticks from each sample were chosen for the μTBS test (*n* = 40 per group). Each stick was fixed on a testing jig installed on a micro-tensile tester (Bisco, Schaumburg, IL, USA) under tension at a 1 mm/min speed until failure. The maximum load (N) was recorded and the precise interface area (mm^2^) was measured by a digital caliper (IP54, Meinaite tools, Shanghai, China) to calculate the bond strength of μTBS value (MPa).

Failure patterns were observed and classified into cohesive failure within the dentin (CD) or composite resin (CC), adhesive failure (A), and mixed failure (M), by stereomicroscope at ×50 magnification (Stemi 2000-C, Carl Zeiss Jena, Gottingen, Germany) [32]. Representative interfacial ultrastructure of adhesive or mixed failure on dentin side was observed in detail using scanning electron microscopy (SEM, FlexSEM 1000, Hitachi, Tokyo, Japan) at 10 kV. Before SEM observation, the specimens were dried for 24 h and coated with gold by an ion sputter coater (108Auto, Ted Pella, Redding, CA, USA; single cycle for 30 s) [23].

### 2.5. Dentin Tubular Sealing Observation

To evaluate the dentin tubular occlusion by desensitizing agents, dentin discs were treated according to the desensitizing protocols in Section 2.2 and observed by SEM at 10 kV under 2000 and 5000 magnifications [23]. The element distribution of dentin tubular occluding deposits was analyzed by energy-dispersive X-ray (EDX) mapping with EDX/WDX system (AZtec X-Max 50, Oxford Instruments, Oxford, UK). The qualitative elemental content (wt %) was performed with standard-less ZAF correction.

To explore the acid etching effect of universal adhesives with etch-and-rinse or self-etch mode on the tubular occlusion of desensitized dentin, dentin discs were treated with desensitizing agents and then bonded using three universal adhesives following the manufacturer’s instructions without light curing, respectively (*n* = 8). The specimens were left standing for 15 s, thoroughly washed by acetone and water for 30 s. The dentin surfaces were dried for 24 h, gold coated and observed by SEM at 10 kV (×5000 magnifications) [23].

### 2.6. Contact Angle

To evaluate the wettability properties of universal adhesives with etch-and-rinse or self-etch mode on desensitized dentin, the sessile drop technique was adopted to measure contact angles by a Contact Angle System (DSA100S, KRUSS, Hamburg, Germany). Dentin discs were treated by desensitizing agents according to the protocols in Section 2.2. Following the manufacturer’s protocols, etch-and-rinse mode specimens were subjected to acid etching by 35% H_3_PO_4_ etchant for 15 s immediately before measurement. Droplets of each universal adhesive liquid in a volume of 3μL were gently dropped onto the dentin substrate after removing excess water through a micro-syringe, respectively (*n* = 8). Static contact angle views were recorded to calculate the angle values [33,34].

### 2.7. Confocal Laser Scanning Microscopy Analysis

To examine the adhesive interfaces of universal adhesives with etch-and-rinse or self-etch modes on desensitized dentin, mid-coronal specimens were subjected to desensitizing application. An amount of 0.1 wt % Rhodamine B (Sigma-Aldrich, St. Louis, MO, USA) was prelabeled on the universal adhesives. According to the manufacturer’s protocols, desensitized specimens were bonded with the fluorescent universal adhesives, restored by 1 mm thick composite resin and cut into slabs longitudinally to the bonding interfaces (*n* = 6). Observed surfaces were wet-polished to 2000-grit, ultrasonically bathed for 10 min and examined by confocal laser scanning microscope (CLSM, FV3000, Olympus, Tokyo, Japan). Fluorescence emission along the adhesive layer and within the dentin tubules was detected using 570 nm wavelengths laser (Rhodamine B excitation). Penetration of resin tags into dentinal tubules was obtained and processed by Image J (NIH, Bethesda, MD, USA).

### 2.8. Nanoleakage Evaluation

Eight residual slabs from each subgroup of the μTBS test were prepared for nanoleakage evaluation. Nail varnish was coated on slabs leaving 1 mm space away from bonding interfaces. Subsequently, slabs were soaked in 50 wt % ammoniacal AgNO_3_ solution (pH = 9.5) at total darkness for 24 h, thoroughly cleaned and immersed in a photo-developing solution for 8 h with fluorescent light irradiation to convert the [Ag(NH_3_)_2_]^+^ into the silver particles. Silver-stained slabs were gently wet-polished by SiC papers in sequence to 2000-grit, immersed in 6% HCl solution for 10 s, 5% NaClO solution for 5 min, ultrasonically cleaned for 10 min, and observed using a FESEM (MAIA3 LMH, Tesca, Brno, Czech Republic) in the backscattered electron mode at 10 kV. Before observation, the specimens were dried for 24 h and coated with gold [23].

### 2.9. Statistical Analysis

Statistical analysis was performed using the SPSS software (24.0, SPSS Inc., Chicago, IL, USA). μTBS values and contact angle values were analyzed by the Shapiro–Wilk test for normal distribution and Levene’s tests for homogeneity of variance before the parametric analysis (*p* > 0.05), then three-factor analysis of variance (ANOVA) was used to estimate the variable effects, including desensitizer types, adhesive types, bonding modes and interaction factors followed by Tukey’s multiple comparison test. Data of resin tag lengths were detected by the non-parametric Kruskal–Wallis test and Mann–Whitney U-test with Bonferroni adjustment due to the data not complying with normality and homogeneity assumptions. For all analyses, statistical significance was pre-set at α = 0.05.

## 3. Results

### 3.1. Micro-Tensile Bond Strength

**Figure 2:**
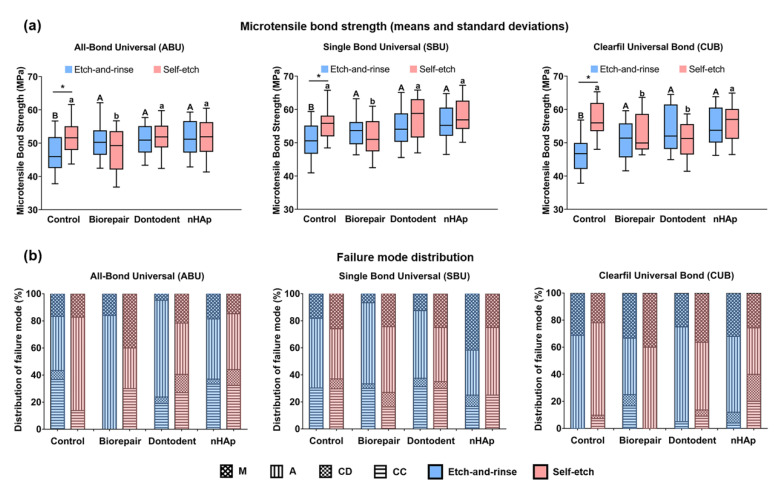
Micro-tensile bond strength and failure mode. (**a**) Effects of desensitizer types and bonding modes on bond strength of universal adhesives. Different superscript uppercase letters indicate significant differences with etch-and-rinse mode (*p* < 0.05). Different superscript lowercase letters indicate significant differences with self-etch mode (*p* < 0.05). * denotes a significant difference between the etch-and-rinse mode and self-etch mode (*p* < 0.05). (**b**) Failure mode distribution of fracture specimens. M: mixed failure; A: adhesive failure; CD: cohesive failure within the dentin; and CC: cohesive failure within the composite resin.

Figure 2a represents μTBS results of all groups. Three variables of desensitizer types (F = 15.65, *p* < 0.001), adhesive types (F = 36.62, *p* < 0.001), and bonding modes (F = 15.29, *p* < 0.001) significantly affected the bond strength. For interactive factors, there was a significant interaction between desensitizer types and bonding modes (F = 20.38, *p* < 0.001). Etch-and-rinse mode showed decreased bond strength of control groups compared with self-etch mode (*p* < 0.01), however, no significant difference between the two modes was found among desensitizing groups (*p* > 0.05). Among the etch-and-rinse mode groups, the control group generated the lowest μTBS compared with Biorepair, Dontodent, and nHAp groups (*p* < 0.05), regardless of the adhesive types. Comparison among desensitizing groups of etch-and-rinse mode revealed no difference (*p* > 0.05). For self-etch mode, Biorepair presented the lowest μTBS bonded with All-Bond Universal and Single Bond Universal, Biorepair and Dontodent reduced μTBS bonded with Clearfil Universal Bond (*p* < 0.05). 

**Figure 3:**
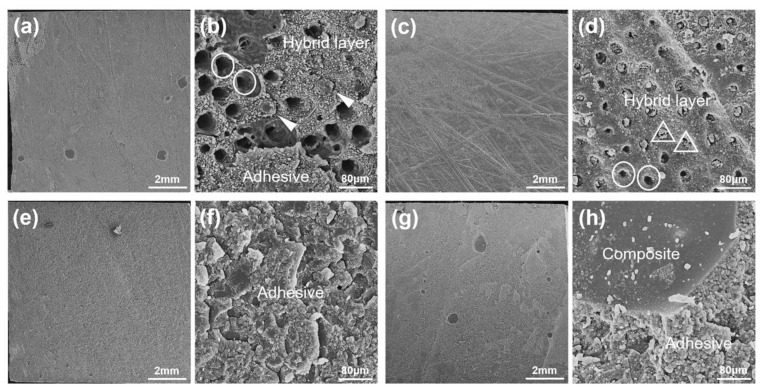
General condition graphs (×90) and micrographs (×2000) of adhesive and mixed failure on dentin interfaces. (**a**,**b**) Mixed failure from control group bonded with etch-and-rinse mode; (**c**,**d**) adhesive failure from desensitizing groups bonded with self-etch mode; (**e**,**f**) typical adhesive failure; and (**g**,**h**) typical mixed failure. Circle: open dentin tubules; triangle: sealed dentin tubules; and solid arrowhead: resin tags.

Figure 2b shows the frequency distribution of fracture patterns. Primary modes were adhesive and mixed patterns for all subgroups. Representative images of adhesive and mixed failure are presented in Figure 3. A mixed failure involved hybrid layer and adhesive layer from the control group bonded with the etch-and-rinse mode is shown in Figure 3a,b. Part of the dentin tubules was unfilled with resin tags at the bottom of the hybrid layer. Adhesive resin poorly infiltrated the intertubular dentin and dentinal tubules. Figure 3c,d presents the adhesive failure at the hybrid layer from desensitizing groups bonded with self-etch mode. Dentinal tubules were widely sealed by unconsolidated desensitizer particles rather than resin tags. Figure 3e,f displays the adhesive failure at the adhesive layer, densely covered by adhesive resin. A classical mixed failure is performed in Figure 3g,h, including adhesive layer and composite.

### 3.2. Dentin Tubular Sealing Observation

**Figure 4:**
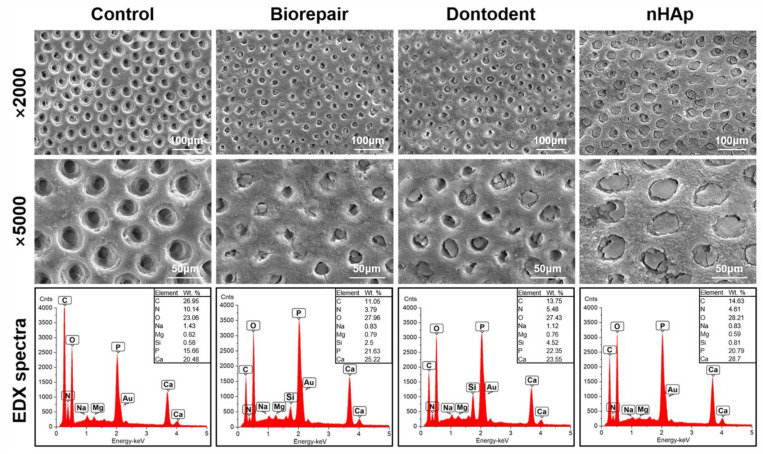
SEM image observation and EDX spectrum of dentin surface after desensitization treatment (×2000 and ×5000).

Dentin surfaces were free of a conspicuous smear layer, and the tubular orifices were all opened in the control group. After desensitization, the dentin surface was partially covered by desensitizer particles; nHAp achieved superior tubular occlusion (Figure 4). EDX analysis showed mineral deficiency with low mineral peaks (Ca and P) and high organic peaks (C, N, and O) in the control group. Silica phases confirmed the presence of silicates in Biorepair and Dontodent dentifrice. Compared with the control group, desensitized groups presented higher Ca peaks and P peaks; the nHAp group noted the highest level of Ca elements. Due to the substantial overlap between the P and Au phases in the spectrum on the gold-coated dentin, the accuracy Ca/P ratio cannot be determined.

**Figure 5:**
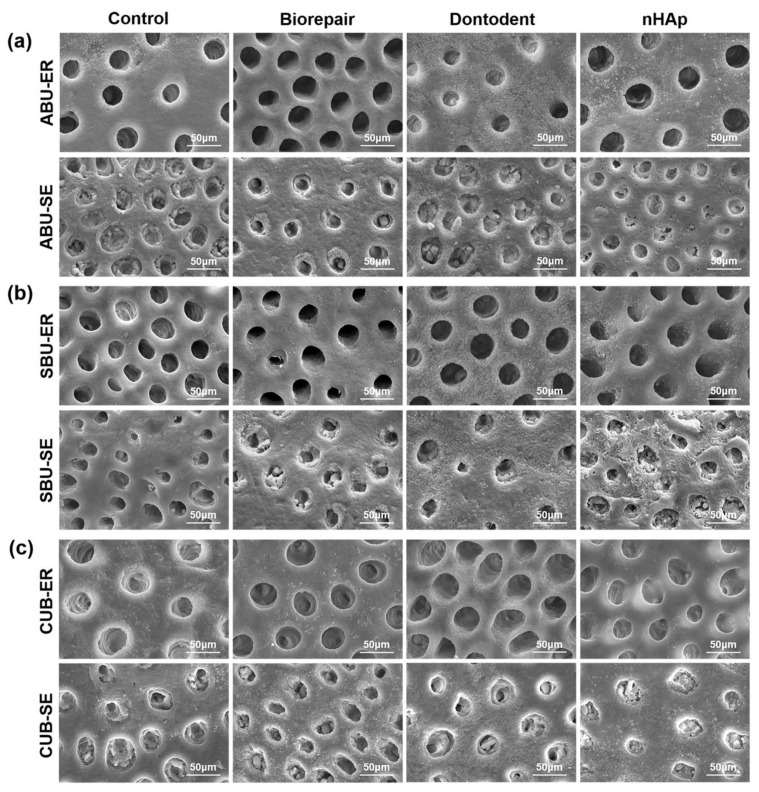
Morphological SEM images of desensitized dentin surfaces treated by universal adhesives with etch-and-rinse mode or self-etch mode. (**a**) All-Bond Universal (ABU). (**b**) Single Bond Universal (SBU). (**c**) Clearfil Universal Bond (CUB). ER: etch-and-rinse mode; SE: self-etch mode.

The morphological change of desensitized dentin surfaces treated by universal adhesives with etch-and-rinse mode or self-etch mode is presented in Figure 5. In phosphoric acid pretreated groups (etch-and-rinse mode), the covered layer was removed, and similar large-scope demineralization with exposed dentinal tubules appeared. Acidic monomers contained in the universal adhesive (self-etch mode) alone partially demineralized dentin surfaces and reduced tubular occlusion to half among all the desensitizing groups.

### 3.3. Contact Angle

**Figure 6:**
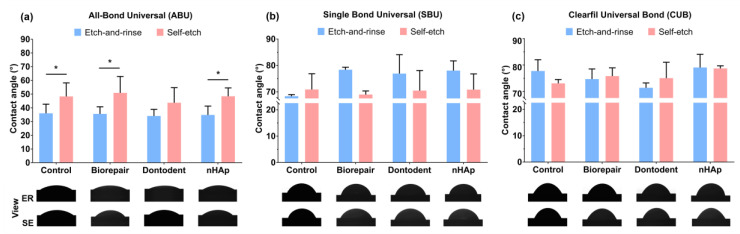
Contact angle values and anterior view of universal adhesives on dentin substrates after desensitization. (**a**) All-Bond Universal. (**b**) Single Bond Universal. (**c**) Clearfil Universal Bond. * denotes a significant difference between the etch-and-rinse mode and self-etch mode (*p* < 0.05).

Contact angle values and anterior view of universal adhesives on dentin after desensitizing treatment are represented in Figure 6. The variables of adhesive types (F = 235.20, *p* < 0.001) and the interaction factors between adhesive types and bonding modes (F = 15.29, *p* < 0.001) significantly affected the contact angles. Desensitization exerted no significant influence on dentin wettability (*p* > 0.05). For All-Bond Universal, lower contact angles were noticed with the etch-and-rinse mode compared with the self-etch mode (*p* < 0.05). There was no significant difference between the etch-and-rinse mode and the self-etch mode bonded with Single Bond Universal and Clearfil Universal Bond (*p* > 0.05). All-Bond Universal showed a significantly lower contact angle than Single Bond Universal and Clearfil Universal Bond (*p* < 0.05).

### 3.4. Confocal Laser Scanning Microscopy Analysis

**Figure 7:**
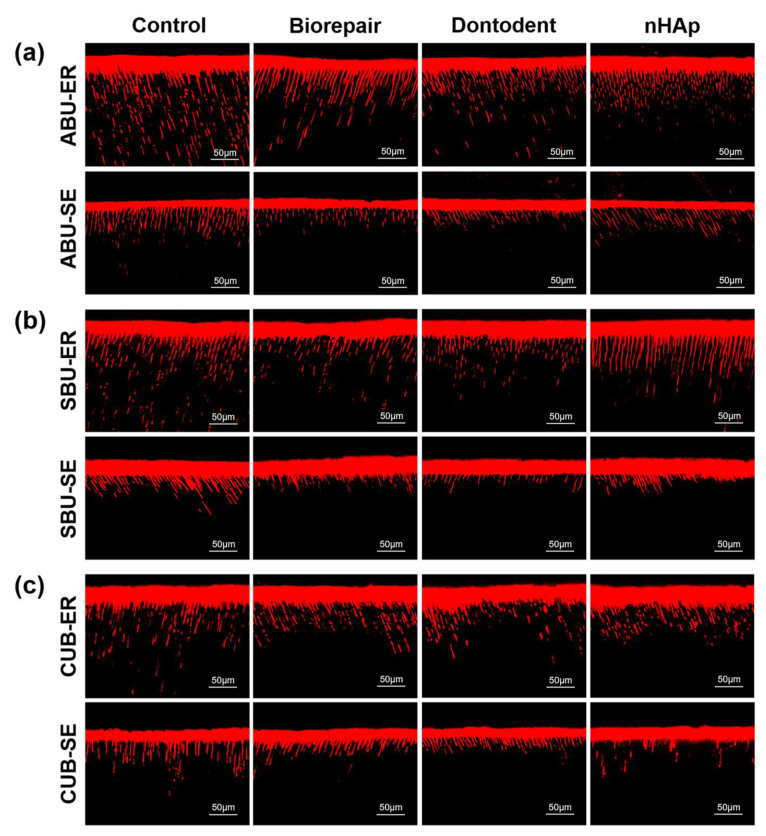
Adhesive resin infiltration observation in etch-and-rinse mode or self-etch mode. (**a**) All-Bond Universal (ABU). (**b**) Single Bond Universal (SBU). (**c**) Clearfil Universal Bond (CUB). Long homogeneous resin tags formed in etch-and-rinse mode and short resin tags formed in self-etch mode. ER: etch-and-rinse mode; SE: self-etch mode.

CLSM displayed adhesive monomers infiltration of universal adhesives with etch-and-rinse mode or self-etch mode (Figure 7). When universal adhesives were applied on phosphoric-acid etched dentin (etch-and-rinse mode), adhesive infiltrated layers were filled with fluorescence dye and long resin tags distributed densely. Shallow adhesive penetration with a low concentration of short resin tags was produced without phosphoric acid treatment (self-etch mode). Control groups revealed more adhesive infiltration into dentin tubules with a higher concentration of long resin tags than desensitizing groups.

**Figure 8:**
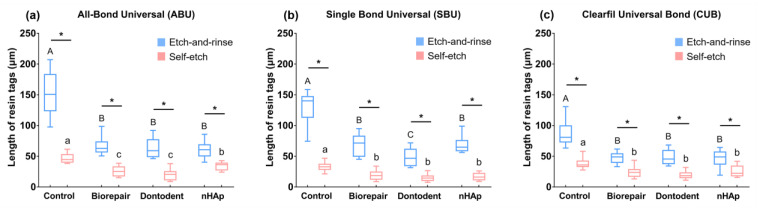
Resin tags’ length of universal adhesives in etch-and-rinse mode or self-etch mode. (**a**) All-Bond Universal. (**b**) Single Bond Universal. (**c**) Clearfil Universal Bond. Different superscript uppercase letters indicate significant differences in etch-and-rinse mode (*p* < 0.05). Different superscript lowercase letters indicate significant differences in self-etch mode (*p* < 0.05). * denotes a significant difference between the etch-and-rinse mode and self-etch mode (*p* < 0.05).

The length of resin tags is shown in Figure 8. Longer resin tags were found in etch-and-rinse mode groups than self-etch mode groups, irrespective of desensitizer and adhesive types (*p* < 0.05). Deeper resin monomer infiltration and longer resin tags were presented in control groups, compared with desensitizing groups (*p* < 0.05).

### 3.5. Nanoleakage Evaluation

**Figure 9:**
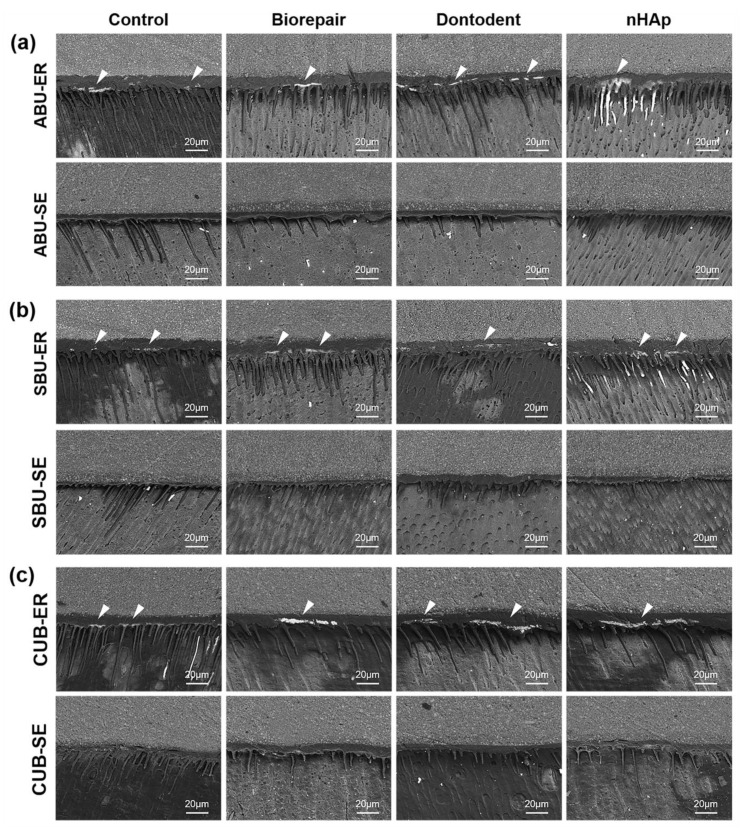
Nanoleakage within the resin–dentin bonding interfaces in etch-and-rinse mode or self-etch mode. (**a**) All-Bond Universal (ABU). (**b**) Single Bond Universal (SBU). (**c**) Clearfil Universal Bond (CUB). A silver-free zone distributed along adhesive interfaces in self-etch mode. Spotted silver deposits were restricted to the hybrid layer of etch-and-rinse mode. Solid arrowhead: nanoleakage indicated by silver deposits. ER: etch-and-rinse mode; SE: self-etch mode.

Illustrative backscattered images of bonding interfaces are shown in Figure 9. For self-etch mode, a silver-free zone was distinct along the hybrid layer and around the resin tags. Conversely, obvious spotted discontinuous infiltration of silver deposits restricted to the bottom of the hybrid layer when bonded with etch-and-rinse mode. For resin tags’ observation, a similar trend is displayed as fluorescence images (Figure 7). The penetration depth of resin tags with etch-and-rinse mode was deeper than that with self-etch mode.

## 4. Discussion

The null hypothesis was partially rejected in this study. More reopened dentin tubules, longer resin tags, and nanoleakage in the resin–dentin interface were observed when using the etch-and-rinse mode than when using the self-etch mode, although no significant difference of bond strength was found between etch-and-rinse mode and self-etch mode on desensitized dentin.

SEM observation in previous studies found that nHAp-based toothpastes can effectively occlude the exposed dentinal tubule to relieve sensitivity [35,36,37]. EDX analysis confirmed that pure nano-sized HA could easily occlude and penetrate into exposed dentinal tubules without disturbance from other toothpaste ingredients. The adhesion mechanism of both etch-and-rinse mode and self-etch mode adhesives are based on the exchange and diffusion process, involving the infiltration of the resin monomer into the demineralized surface and subsequent in situ polymerization [1]. Therefore, sealed dentinal tubules would likely impede adhesive interface formation and bond strength for either bonding mode. However, μTBS values of all desensitizing groups did not show a consistent descending trend, which may have to do with several other aspects influencing bond strength.

Adhesive spread on dentin surface is regarded as an intuitive manifestation of the dentin wettability of adhesive, which can be characterized by contact angle values [38]. Our contact angle results indicated that desensitization did not affect the dentin wettability of all universal adhesives; both of the two bonding modes also exhibited similar contact angles except when used by All-Bond Universal. Phosphoric acid etching (etch-and-rinse mode) can reduce the surface free energy of dentin surface due to a large quantity of HA crystallites dissolution, presented as low wettability, and high contact angle [39,40]. Meanwhile, phosphoric acid also increases dentin surface roughness, which can improve surface wettability and reduce contact angle based on Cassie–Baxter modeling [41]. The contact angle of the etch-and-rinse mode should be consistent with that of the self-etch mode due to the two above contrary factors. In addition, phosphoric acid etching may increase hydrophilicity and decrease the apolar constituent of the dentin surface [42]. All-Bond Universal contains fewer hydrophobic monomers or fillers than Single Bond Universal and Clearfil Universal Bond, such as BisGMA and silane. Ether bonds and hydroxyl bonds of polarity monomers in All-Bond Universal may interact more easily with the dentin surface without the interfere of hydrophobic monomers. Therefore, contact angles were improved by phosphoric acid etching (etch-and-rinse mode) when applying All-Bond Universal.

For self-etch mode, the separate etching steps were replaced by specific acidic functional monomers in universal adhesives. As soon as adhesives were applied on the dentin surface, acid etching and adhesive resin penetration dominated by acidic monomers had already begun simultaneously [4]. Hindered resin infiltration (SEM in Figure 5) and (CLSM in Figure 7) showed that nHAp-based desensitizers significantly interfered with demineralization by acid functional monomers without phosphoric acid etching. Meanwhile, deficient micromechanical interaction, presented as widely blocked dentinal tubules on a fracture surface in desensitizing groups (Figure 3d), may be related to the low bond strength of desensitizing toothpaste groups with self-etch mode.

In addition to micromechanical interlocking, the self-etch mode bond strength is also derived from chemical bonding [1]. Partially demineralized dentin surface with self-etch mode can establish a HAp-rich submicron hybrid layer [1,43]. Retained HA component and nHAp-based desensitizing agents could offer chemical receptors for functional monomers, especially for 10-MDP. 10-MDP is one of the most effective functional monomers with a methacrylate functional group at one monomer end and a hydrophilic phosphoric-acid ester functional group at the other end. The methacrylate functional group helps the monomer polymerization into the adhesive network. Phosphoric-acid ester group can not only produce an etching effect and generate micromechanical interlocking, but also release a large amount of Ca^2+^ from the dental minerals and bond to Ca^2+^ ionically forming a 10-MDP-Ca stable self-assembled nano-layer [1]. Based on the EDX analysis, pure nHAp may provide more potential calcium sites for chemical reactions with 10-MDP to compromise the adverse effects on the bond strength. Therefore, pure nHAp could explain higher bond strength than nHAp-based toothpastes, which was in agreement with previous reports [9,13,44]. 

For the etch-and-rinse mode, the bond strength was found similar to self-etch mode on desensitized dentin, in accordance with several studies on normal dentin [3,4,45,46]. Desensitizer particles on dentin surface could be completely neutralized by phosphoric acid and acidic functional monomers, performed as a similar demineralized image with the control group (Figure 5). When using the same universal adhesive, it might be difficult for the etch-and-rinse mode to form a chemical bond on the hybrid layer with less retained HA and nHAp-based agents compared with the self-etch mode. Meanwhile, resin monomers in universal adhesive can penetrate further into the entirely reopened dentinal tubule (etch-and-rinse mode) and offer more micromechanical bond strength than self-etch mode, based on CLSM observation ( Figure 7 and Figure 8). Therefore, the etch-and-rinse mode showed bonding strength close to that of the self-etch mode on desensitized dentin.

Based on μTBS results, the lowest bond strength in the control group bonded with etch-and-rinse mode may be attributed to excessive dentin acid etching. According to the adhesion–decalcification (A–D) concept, phosphoric acid is an effective etchant, causing apatite crystallite dissolution and collagen fibrils exposure in 5–8 μm depth demineralized region [1,3,39].When applying etch-and-rinse mode, phosphoric acid was applied prior to the adhesives, and acidic functional monomers in universal adhesives might cause dentin over-etch on the pre-etched surface by phosphoric acid [47].

In general, low pH (pH < 2) universal adhesives with etch-and-rinse mode might produce aggressive erosion to dentin [3,47]. Universal adhesives in this study are classified as (ultra) mild adhesives, which are unlikely to trigger immoderate acid etching for normal dentin. However, for sensitive dentin, phosphoric acid may initiate deeper demineralization along the exposed dentin tubules. Resin monomer can hardly infiltrate down to the demineralized depth, resulting in overexposure of collagen fibers and hybrid layer degradation [1,3,4]. The etch-and-rinse strategy was always technique-sensitive for dentin bonding. According to the wet-bonding technique, over-etch makes the collagen network more likely to collapse and prevents resin monomers from penetrating into the demineralized region [1]. Our fracture pattern findings showed that poor resin infiltration into intertubular dentin and dentinal tubules appeared in the control group with etch-and-rinse mode (Figure 3b). 

Nanoleakage is accepted as an essential indicator to evaluate adhesive’s sealing capability and bonding effectiveness [48]. Resin infiltration failure or unsubstituted residual water causes nanoleakage within the hybrid layer. Nanoleakage was represented as silver nitrate deposition and served as the starting point of water and endogenous enzyme degradation [3,4]. Increased silver infiltration along the adhesive interface with etch-and-rinse mode (Figure 9) may be attributed to the inconsistent depth between resin penetration and demineralization. As the depth of acid etching increases, it may be difficult for resin monomers to penetrate the depth of dentin demineralization. 

Based on our results, clinicians may consider using nHAp-based desensitizing toothpastes as an ideal treatment option for DH. However, its adverse influence on the bond strength should not be ignored when universal adhesives were applied. This study had some limitations, including in vitro design without oral environmental conditions such as temperature change, pH cycling variation, masticatory stress load, and lack of aging degradation of the bonding test. Further studies should be conducted to evaluate the long-term bonding durability of these adhesives, and clinical research is required to evaluate the actual longevity of universal adhesives to provide much more clinical guidance for dentists.

## 5. Conclusions

Based on the study, the following conclusions were drawn:

1. nHAp-based desensitizing agents compromise the bond strength and adhesive resin penetration of universal adhesives with self-etch mode.
2. Compared with self-etch mode, the etch-and-rinse technique produces comparable bond strength on nHAp-based desensitized dentin, but increases nanoleakage of the adhesive interfaces.

## Figures and Tables

**Table 1:** Composition and application protocols of universal adhesives with etch-and-rinse and self-etch mode according to the manufacturer’s instructions.

Adhesive	pH	Basic Compositions	Etch-and-Rinse Strategy	Self-Etch Strategy
All-Bond Universal (Bisco, USA)	3.2	10-MDP, HEMA, Bis-GMA, ethanol, water, photoinitiators	1. Apply etchant for 15 s; 2. Rinse thoroughly with water spray and remove excess water by air-drying or blotting with cotton pellets; leaving the surface moist; 3. Apply adhesive as the self-etch mode.	1. Apply two separate coats of adhesive and scrub for 10–15 s per coat; 2. Evaporate excess solvent by thoroughly air-drying for at least 10 s until there is no visible movement of the adhesive; 3. The surface should have a uniform glossy appearance; otherwise, apply an additional coat of adhesive and repeat Step 2; 4. Light cure for 10 s.
Single Bond Universal (3M ESPE, USA)	2.7	10-MDP, HEMA, Bis-GMA, DCDMA, MPTMS, VitrebondTM copolymer, silane, ethanol, water, photoinitiators	1. Apply etchant for 15 s; 2. Rinse thoroughly with water spray and dry and remove excess water by water- and oil-free air or blotting with cotton pellets; leaving moist; 3. Apply adhesive as the self-etch mode.	1. Apply the adhesive and rub for 20 s; 2. Evaporate solvent by gently air-drying for approximately 5 s; 3. Light cure for 10 s.
Clearfil Universal Bond Quick (Kuraray Noritake Dental, Japan)	2.3	10-MDP, 2-HEMA, Bis-GMA, hydrophilic amide methacrylate, MPTMS, NaF, colloidal silica, silane coupling agent, photoinitiators	1. Apply etchant, leave it in place for 15 s, then rinse and dry; 2. Apply adhesive as the self-etch mode.	1. Apply the adhesive with a rubbing motion without waiting time; 2. Dry the surface sufficiently by blowing mild air for more than 5 s until the adhesive does not move; 3. Light-cure for 10 s.

10-MDP: 10-methacryloyloxydecyl dihydrogen phosphate methacrylate; bis-GMA: bisphenol-A-diglycidyl; HEMA: hydroxyethyl methacrylate; DCDMA: decamethylene dimethacrylate; MPTMS: γ-methacryloxypropyl trimethoxysilane; and VitrebondTM copolymer: methacrylate-modified polyalkenoic acid copolymer.

## Data Availability

The data presented in this study are available on request from the corresponding author.
